# Spectrum of lithium induced thyroid abnormalities: a current perspective

**DOI:** 10.1186/1756-6614-6-3

**Published:** 2013-02-07

**Authors:** Davis Kibirige, Kenneth Luzinda, Richard Ssekitoleko

**Affiliations:** 1 Department of Medicine, Uganda Martyrs Hospital Lubaga, Kampala, Uganda; 2 Department of Medicine, Makerere University College of Health Sciences, Kampala, Uganda

**Keywords:** Lithium therapy, Thyroid abnormalities, Goitre, Hypothyroidism, Hyperthyroidism, Thyroid autoimmunity

## Abstract

**Background:**

Lithium is an integral drug used in the management of acute mania, unipolar and bipolar depression and prophylaxis of bipolar disorders. Thyroid abnormalities associated with treatment with lithium have been widely reported in medical literature to date. These include goitre, hypothyroidism, hyperthyroidism and autoimmune thyroiditis. This current review explores the varied thyroid abnormalities frequently encountered among patients on lithium therapy and their management, since lithium is still a fundamental and widely drug used in psychiatry and Internal Medicine.

**Methods:**

PubMed database and Google scholar were used to search for relevant English language articles relating to lithium therapy and thyroid abnormalities up to December 2012. The search terms used were lithium treatment, thyroid abnormalities, thyroid dysfunction, goitre, hypothyroidism, hyperthyroidism, thyrotoxicosis, autoimmune thyroiditis, lithium toxicity, treatment of affective disorders and depression and side effects of antipsychotic drugs. Reference lists of the identified articles were further used to identify other studies.

**Results:**

Lithium affects normal thyroid functioning through multiple mechanisms. At the cellular level, it decreases thyroid hormone synthesis and release. It also decreases peripheral deiodination of tetraiodothyronine (T4) or thyroxine by decreasing the activity of type I 5’ de-iodinase enzyme. Hypothyroidism and goitre (clinically and/ultrasonographically detected) are the most prevalent thyroid abnormalities among patients on long term lithium therapy. Lithium induced hyperthyroidism is very infrequent. Lithium increases the propensity to thyroid autoimmunity in susceptible individuals due to its effect of augmenting the activity of B lymphocytes and reducing the ratio of circulating suppressor to cytotoxic T cells.

**Conclusions:**

Thyroid function tests (serum thyroid stimulating hormone, free thyroid hormones-T_4_ and triiodothyronine [T_3_] concentrations and thyroid auto-antibodies) and assessment of thyroid size clinically and by thyroid ultrasonography ought to be performed among patients initiating lithium therapy at baseline and later annually. More frequent assessment of thyroid function status and size during the course of therapy is recommended among middle aged females (≥50 years), patients with a family history of thyroid disease and those positive for thyroid auto-antibodies (anti-thyroid peroxidase and TSH receptor antibodies).

## Introduction

Lithium remains an imperative drug in the long term therapy of bipolar affective disorders. It is also a proven prophylactic agent against relapses or recurrences of abnormal mood episodes in unipolar depression, hypomania and mania [1,2]. It has also been shown to reduce suicidal risk and short term mortality [3].

Despite its proven efficaciousness, its use is associated with a myriad of clinical shortcomings. These include: a narrow therapeutic window hence necessitating regular monitoring of therapeutic concentrations, cardiac toxicity, renal tubular dysfunction and endocrinopathies like thyroid abnormalities, hyperparathyroidism, transient hyperglycemia and nephrogenic diabetes insipidus [2,4-7]. This review will focus mainly on the effects of lithium on the normal physiological functioning of the thyroid gland and the frequently reported thyroid abnormalities associated with lithium therapy.

### General pharmacological features of lithium

Lithium is an alkali metal which is available mainly as lithium carbonate and citrate in immediate- and sustained-release preparations. It reaches peak plasma concentrations in 1–2 and 4–5 hours for the immediate and sustained release formulations respectively with an elimination half life of 18–36 hours. Its excretion is primarily via the kidneys and this renal clearance decreases with increasing age [8].

The precise mechanisms by which lithium exerts its mood stabilising effects are still not very apparent. Its neurotropic effects are partially explained by the inhibitory effect on the N-methyl D-aspartate receptor that mediates cellular calcium influx and the suppression of activation of pro-apoptotic calcium dependent signalling pathways [9]. Lithium also alters release of neurotransmitters and lessens glutaminergic activity [10].

### Effects of lithium on the physiology of the thyroid gland

Multiple effects of lithium on the physiology of the thyroid gland have been extensively studied. Lithium has been shown to be highly concentrated in thyroid cells. In-vivo and vitro studies in rats have shown that lithium reduces the uptake of radioiodine into rat thyroid and salivary glands. In humans, lithium administration may result in either reduced or increased thyroidal radioiodine uptake. Several mechanisms are thought to explain this dual effect among humans. Low thyroid iodine uptake could be due to lithium induced iodide retention and competition for the iodide transport within the thyroid gland. An increase in the uptake could be mediated by the increased secretion of thyroid stimulating hormone (TSH) following lithium induced hypothyroidism [11].

Another key effect of lithium on thyroid gland functioning occurs at the level of hormone synthesis and release. Lithium inhibits synthesis and release of thyroid hormones. This inhibitory effect is due to the alteration in the tubulin polymerisation and inhibition of the action of TSH on cyclic adenosine mono phosphate (c-AMP). Lithium also alters the structure of thyroglobulin thereby affecting protein conformation and function with subsequent iodotyrosine coupling defects. Lithium administration is associated with reduced hepatic deiodination and clearance of free thyroxine (T_4_). The latter induces a decrease in the activity of type I 5’ de-iodinase enzyme [11,12].

### Thyroid abnormalities due to lithium therapy

#### Goitre

The initial inhibition of thyroid hormone synthesis and release by lithium results into increased TSH concentrations leading to thyroid enlargement. Other mechanisms proposed to explain thyrocyte proliferation among patients on lithium therapy are activation of the pro-proliferative tyrosine kinase and Wnt/beta-catenin signalling pathways [12-14].

Goitre is the most common clinical finding noted among patients on lithium therapy. It occurs as a diffuse and non tender neck swelling. Varying prevalence of goitre among treated patients has been reported in the several published studies. This is explained by the differences in iodine content in the geographical study settings, duration of lithium use and diagnostic study techniques used [12].

In one early study by Schou et al. among 330 manic-depressive patients on lithium therapy, clinically detected goitre was noted among 12 patients, giving a prevalence of 3.6%. The calculated annual incidence was 4% compared to 1% incidence in a geographically comparable healthy general population [15]. In another survey by Bocchetta et al. among 150 outpatients on long term lithium therapy, the prevalence of a visible and / clinically palpable goitre was 51% [16]. This prevalence reduced with subsequent follow up [17,18].

Thyroid ultrasonography has been demonstrated to be a simple, cheap and sensitive method for screening for goitre and thyroid abnormalities among patients on lithium therapy [19]. In one cross sectional study to determine the thyroid size and prevalence of goitre among 96 treated patients with affective disorders in Germany, goitre was reported among 53 (55%) patients on lithium therapy and 19 (20%) controls (p = 0.003) [20]. In this study, screening using thyroid ultrasonography identified more patients with goitre compared to clinical palpation (N = 53 Vs 19, p < 0.01 compared to N = 24 Vs 12, p < 0.001 respectively).

Other cross sectional studies have reported similar findings of higher frequency of goitre of 50% [21]-59% [22] among lithium treated patients.

The approach to management of lithium induced goitre is comparable to that among healthy population. However, levothyroxine replacement therapy is preferred more among patients with significant thyroid enlargement and accompanying compressive symptoms [12].

#### Hypothyroidism and subclinical hypothyroidism

Hypothyroidism and subclinical hypothyroidism with/without concomitant goitre is also very prevalent among patients on lithium therapy in varying reported frequencies ranging from 0% to 52% [16,22-27]. Similarly, disparities in prevalence can be explained by different study definitions, iodine intake and baseline thyroid autoimmunity among study subjects [28]. The average duration of lithium administration prior to the diagnosis of hypothyroidism is about 18 months, although it can occur within the first few months [29].

Bocchetta et al. in a study among 150 ambulatory Sardinian patients on lithium therapy, no cases of clinical hypothyroidism were documented. Subclinical hypothyroidism was noted in 19% of the patients. Among lithium treated patients with positive thyroid antibodies, the prevalence of subclinical hypothyroidism increased to 53%. The prevalence of specific antithyroid antibodies was positively correlated with age and duration of lithium treatment, and was higher among women [16].

In another cross sectional study by Kirov et al. among 115 males and 159 females with affective disorders on long term lithium therapy in the UK, the prevalence of hypothyroidism was 10.3%. The prevalence was higher among female patients compared to males (n = 27, 17% Vs n = 4, 3.5% respectively) [30]. A retrospective study of 718 patients on lithium therapy by Johnson and Eagles reported a comparable prevalence of clinical hypothyroidism of 10.4% especially among female gender (14% Vs 4.5%). The calculated annual incidence of hypothyroidism in this study was 2.17% and 0.68% among women and men respectively [26], relatively higher than the reported figures among the community in the Whickham survey [31].

The aetiology of lithium associated hypothyroidism and subclinical hypothyroidism is primarily related to inhibition of synthesis and release of thyroid hormones [12]. Other studies have demonstrated a transient lithium induced increase in titres of thyroid auto-antibodies (thyroid peroxidase auto-antibodies) present prior to lithium administration. No lithium induced production of thyroid auto-antibodies independently is thought to occur [32,33]. However, some studies have reported contrasting results in relation to lithium inducing an increase in the titres of thyroid auto-antibodies. In a cross sectional study by Baethge et al. among 100 adults with major affective disorders matched with 100 age-and sex matched controls with no history of an axis I psychiatric diagnosis, there was no significant difference in the prevalence of auto-antibodies against thyroid peroxidase and thyroglobulin between the patients and the age- and sex matched healthy controls (0.07% Vs 0.11% and 0.08% Vs 0.15% respectively). TSH receptor auto-antibodies were not found in either group [34].

The risk of developing lithium induced hypothyroidism has been shown to be significantly higher among females, with increasing age (>50 years), presence of family history of thyroid disease and thyroid auto-antibodies [28,29,35]. Due to this high frequency of hypothyroidism, it is clinically plausible to assess the thyroid function status, thyroid size and presence of thyroid auto-antibodies in all patients prior to initiation of lithium therapy and later annually. More frequent assessment of thyroid function tests (at least every 3–4 months) among middle aged females (>50 years) who are thyroid auto-antibody positive with family history of thyroid disease is recommended [29,36]. Levothyroxine replacement therapy concurrently with lithium administration especially in the presence of clinically overt hypothyroidism, significantly enlarged thyroid glands, subclinical hypothyroidism and in rapidly cycling or treatment resistant cases is recommended in the management of lithium induced hypothyroidism [29].

#### Hyperthyroidism/thyrotoxicosis

Hyperthyroidism may occur with long term lithium treatment though to a less extent compared to goitre and hypothyroidism [23,30,37-40]. In the retrospective study by Kirov among 209 patients with affective disorders on long term lithium therapy, post treatment thyrotoxicosis was noted among 2 patients (1 male and 1 female) [23]. Thyrotoxicosis however was noted to occur earlier in the course of treatment and at a younger age in the female patient compared to the male patient (6 years Vs 9 years later and 42 years Vs 50 years respectively).

In another cross sectional study, 2 (1.8%) of 111 men who had a mean duration of 80 months of lithium therapy developed thyrotoxicosis, giving an incidence rate of 2.7/1000 survivor years. Only 6 (3.9%) of 152 women with a mean duration of 73.3 months of lithium therapy developed thyrotoxicosis, giving an incidence rate of 6.4/1000 survivor years [24]. In the prospective study by the same research group among 33 women, only 1 woman developed hyperthyroidism over the 146 person-years of follow up [24]. Bocchetta et al. noted only 1 case of hyperthyroidism among 150 patients on long term lithium therapy during a 15 year follow up equivalent to 976 patient-years, suggesting that lithium induced hyperthyroidism is extremely rare [40].

Lithium induced hyperthyroidism is mainly characterised by a transient and painless thyroiditis. Some published reports have also shown that lithium is associated with granulomatous thyroiditis, lymphocytic thyroiditis or non-specific thyroiditis [41,42]. In one single centre experience among 23 patients with lithium induced thyrotoxicosis with a median duration of 6 years of lithium therapy (0.6-25 years), transient thyrotoxicosis with a painless thyroiditis was noted among 9 (23%) patients [42].

This transient and painless thyroiditis is thought to be due to a possible direct toxic effect of lithium on the thyroid gland [41]. Another mechanism proposed in the pathogenesis of lithium induced hyperthyroidism/thyrotoxicosis is related to autoimmunity and auto-antibody production. In a study by Wilson et al., 20% of lithium treated patients had thyroid auto-antibodies compared to 7.5% not on lithium treatment. Lithium treatment in this study was shown to increase B cell activity and decreased ratios of suppressor to cytotoxic T cells [43]. This demonstrates that lithium induces thyroid autoimmunity among susceptible individuals.

Patients with lithium induced hyperthyroidism are best treated with anti thyroid drugs like carbimazole with/without steroids. Radioiodine or thyroidectomy should be reserved for patients with lithium induced Graves’ disease especially in cases of poor compliance to the anti thyroid drugs. In cases of a toxic nodular goitre with/without compressive symptoms, a thyroidectomy is indicated.

In cases of lithium induced thyroiditis, ablative radioiodine is not indicated because of its poor uptake. Conservative management with regular follow up is recommended in such cases since majority of the patients develop hypothyroidism subsequently [42].

## Conclusions

Lithium being an effective and pivotal drug in the management of affective disorders, concomitant thyroid dysfunction remains a pertinent clinical subject to address. Significant proportions of patients treated with lithium develop clinically or radiologically confirmed goitre and hypothyroidism. Lithium increases the risk of thyroid autoimmunity in susceptible individuals. Lithium induced hyperthyroidism is infrequent.

Baseline and regular assessment of thyroid function tests (TSH, free T4); thyroid size using thyroid ultrasonography and measurement of titres of auto-antibodies against thyroid peroxidase is recommended among patients prior and during lithium therapy.

## Competing interests

The authors declare that they have no competing interests.

## Authors’ contributions

DK, KL and RS equally contributed to the development of the concept and manuscript, critically read and approved the final manuscript.

## References

[B1] Geddes J Burgess S Hawton K Jamison K Goodwin G Long-term lithium therapy for bipolar disorder: systematic review and meta-analysis of randomized controlled trials Am J Psychiatry 2004 161 217 222 10.1176/appi.ajp.161.2.217 14754766

[B2] Freeman M Freeman S Lithium: clinical considerations in internal medicine Am J Med 2006 119 478 481 10.1016/j.amjmed.2005.11.003 16750958

[B3] Cipriani A Pretty H Hawton K Geddes J Lithium in the prevention of suicidal behavior and all-cause mortality in patients with mood disorders: a systematic review of randomized trials Am J Psychiatry 2005 162 1805 1819 10.1176/appi.ajp.162.10.1805 16199826

[B4] Tredget J Kirov A Kirov G Effects of chronic lithium treatment on renal function J Affect Disord 2010 126 436 440 10.1016/j.jad.2010.04.018 20483164

[B5] Bhuvaneswar C Baldessarini J Harsh V Alpert J Adverse endocrine and metabolic effects of psychotropic drugs: selective clinical review CNS Drugs 2009 23 12 1003 1021 10.2165/11530020-000000000-00000 19958039

[B6] Giusti C Amorim S Guerra R Portes E Endocrine disturbances related to the use of lithium Arq Bras Endocrinol Metab 2012 56 3 153 158 10.1590/S0004-27302012000300001 22666729

[B7] McKnight R Adida M Budge K Stockton S Goodwin G Geddes J Lithium toxicity profile: a systematic review and meta-analysis Lancet 2012 379 721 728 10.1016/S0140-6736(11)61516-X 22265699

[B8] Grandjean E Aubry J Lithium: updated human knowledge using an evidence-based approach. Part II: clinical pharmacology and therapeutic monitoring CNS Drugs 2009 23 4 331 349 10.2165/00023210-200923040-00005 19374461

[B9] Chiu C Chuang D Molecular actions and therapeutic potential of lithium in preclinical and clinical studies of CNS disorders Pharmacol Ther 2010 128 2 281 304 10.1016/j.pharmthera.2010.07.006 20705090 PMC3167234

[B10] Jope R Anti-bipolar therapy: mechanism of action of lithium Mol Psychiatry 1999 4 2 117 128 10.1038/sj.mp.4000494 10208444

[B11] Lazarus J The effects of lithium therapy on thyroid and thyrotropin-releasing hormone Thyroid 1998 8 10 909 913 10.1089/thy.1998.8.909 9827658

[B12] Lazarus J Lithium and thyroid Best Pract Res Clin Endocrinol Metab 2009 23 723 733 10.1016/j.beem.2009.06.002 19942149

[B13] Urabe M Hershman J Pang X Murakami S Sugawara M Effect of lithium on function and growth of thyroid cells in vitro Endocrinology 1991 129 807 814 10.1210/endo-129-2-807 1649747

[B14] Rao A Kremenevskaja N Resch J Brabant G Lithium stimulates proliferation in cultured thyrocytes by activating Wnt/B-catenin signaling Eur J Endocrinol 2005 153 929 938 10.1530/eje.1.02038 16322400

[B15] Schou M Amdisen A Jensen S Olsen T Occurrence of goiter during lithium treatment Br Med J 1968 3 710 713 10.1136/bmj.3.5620.710 17016877 PMC1989588

[B16] Bocchetta A Bernardi F Pedditzi M Thyroid abnormalities during lithium treatment Acta Psychiatr Scand 1991 83 193 198 10.1111/j.1600-0447.1991.tb05523.x 1903238

[B17] Bocchetta A Cherchi A Loviselli A Six-year follow-up of thyroid function during lithium treatment Acta Psychiatr Scand 1996 94 45 48 10.1111/j.1600-0447.1996.tb09823.x 8841676

[B18] Bocchetta A Mossa P Velluzzi F Mariotti S Del-Zompo M Loviselli A Ten-year follow-up of thyroid function in lithium patients J Clin Psychopharmacol 2001 21 594 598 10.1097/00004714-200112000-00009 11763007

[B19] Loviselli A Bocchetta A Mossa P Value of thyroid echography in the long-term follow-up of lithium-treated patients Neuropsychobiology 1997 36 37 41 10.1159/000119358 9211443

[B20] Bauer M Blumentritt H Reinhard F Using ultrasonography to determine thyroid size and prevalence of goiter in lithium-treated patients with affective disorders J Affect Disord 2007 104 45 51 10.1016/j.jad.2007.01.033 17346802

[B21] Perrild H Hegedüs L Baastrup P Kayser L Kastberg S Thyroid function and ultrasonically determined thyroid size in patients receiving long-term lithium treatment Am J Psychiatr 1990 147 1518 1521 2221166 10.1176/ajp.147.11.1518

[B22] Ozpoyraz N Tamam L Kulan G Thyroid abnormalities in lithium-treated patients Adv Ther 2002 19 176 184 10.1007/BF02848693 12431043

[B23] Kirov G Thyroid disorders in lithium-treated patients J Affect Disord 1998 50 33 40 10.1016/S0165-0327(98)00028-7 9716277

[B24] Kirov G Tredget J John R Owen M Lazarus J A cross-sectional and a prospective study of thyroid disorders in lithium-treated patients J Affect Disord 2005 87 313 317 10.1016/j.jad.2005.03.010 16051025

[B25] Fagolini A Kupfer D Scott J Hypothyroidism in patients with bipolar disorder treated primarily with lithium Epidemiol Psichiatr Soc 2006 15 123 127 16865933 10.1017/s1121189x00004322

[B26] Johnston A Eagles J Prevalence and risk factors-lithium-associated clinical hypothyroidism Br J Psychiatry 1999 175 336 339 10.1192/bjp.175.4.336 10789300

[B27] Aliasgharpour M Abbassi M Shafaroodi H Subclinical hypothyroidism in lithium-treated psychiatric patients in Tehran, Islamic Republic of Iran East Mediterr Health J 2005 11 329 333 16602451

[B28] Bocchetta A Loviselli A Lithium treatment and thyroid abnormalities Clin Pract Epidemiol Ment Health 2006 2 23 10.1186/1745-0179-2-23 16968542 PMC1584230

[B29] Chakrabarti S Thyroid functions and bipolar affective disorder J Thyroid Res 2011 10 4061 10.4061/2011/306367 PMC3144691 21808723

[B30] Kirova G Tredget J John R Owen M Lazarus J A cross-sectional and a prospective study of thyroid disorders in lithium-treated patients J Affect Disord 2005 87 313 317 10.1016/j.jad.2005.03.010 16051025

[B31] Vanderpump M Tunbridge W French J The incidence of thyroid disorders in the community: a twenty-year follow-up of the Whickham Survey Clin Endocrinol (Oxf) 1995 43 55 68 10.1111/j.1365-2265.1995.tb01894.x 7641412

[B32] Weetman A McGregor A Lazarus J Rees-Smith B Hall R The enhancement of immunoglobulin synthesis by human lymphocytes with lithium Clin Immunol Immunopathol 1982 22 400 407 10.1016/0090-1229(82)90057-5 6286192

[B33] Wllson R McKIllop J Crocket G The effect of lithium therapy on parameters thought to be involved in the development of autoimmune thyroid disease Clin Endocrinol 1991 34 357 361 10.1111/j.1365-2265.1991.tb00305.x 2060144

[B34] Baethge C Blumentritt H Berghöfer A Long-term lithium treatment and thyroid antibodies: a controlled study J Psychiatry Neurosci 2005 30 6 423 427 16327876 PMC1277025

[B35] Bocchetta A Bernardi F Burrai C The course of thyroid abnormalities during lithium treatment – a 2-year follow-up study Acta Psychiatr Scand 1992 86 38 41 10.1111/j.1600-0447.1992.tb03222.x 1414397

[B36] Livingstone C Rampes H Lithium: a review of its metabolic adverse effects J Psychopharmacol 2006 20 3 347 355 16174674 10.1177/0269881105057515

[B37] Bandyopadhyay D Nielsen C Lithium-induced hyperthyroidism, thyrotoxicosis and mania: a case report Q J Med 2012 105 83 85 10.1093/qjmed/hcq234 21177317

[B38] Barclay M Brownlie B Turner J Wellst J Lithium associated thyrotoxicosis: a report of 14 cases, with statistical analysis of incidence Clin Endocrinol 1994 40 759 764 10.1111/j.1365-2265.1994.tb02509.x 8033366

[B39] Lazarus J Richards A Adison G Owen G Treatment of thyrotoxicosis with lithium carbonate Lancet 1974 2 1160 1163 4139588 10.1016/s0140-6736(74)90808-3

[B40] Bocchetta A Cocco F Velluzzi F Del-Zompo M Mariotti S Loviselli A Fifteen-year follow-up of thyroid function in lithium patients J Endocrinol Invest 2007 30 5 363 366 17598966 10.1007/BF03346311

[B41] Miller K Daniels G Association between lithium use and thyrotoxicosis caused by silent thyroiditis Clin Endocrinol 2001 55 501 508 10.1046/j.1365-2265.2001.01381.x 11678833

[B42] Brownlie B Turner J Lithium associated thyrotoxicosis Clin Endocrinol 2011 75 402 407 10.1111/j.1365-2265.2011.04048.x 21521317

[B43] Wilson R McKillop J Crocket G The effect of lithium therapy on parameters thought to be involved in the development of autoimmune thyroid disease Clin Endocrinol (Oxf) 1991 34 357 361 10.1111/j.1365-2265.1991.tb00305.x 2060144

